# Swin-cryoEM: Multi-class cryo-electron micrographs single particle mixed detection method

**DOI:** 10.1371/journal.pone.0298287

**Published:** 2024-04-09

**Authors:** Kun Fang, JinLing Wang, QingFeng Chen, Xian Feng, YouMing Qu, Jiachi Shi, Zhuomin Xu

**Affiliations:** 1 Hunan Meteorological Information Center, Hunan Meteorological Bureau, Changsha, Hunan, China; 2 Hunan Key Laboratory of Meteorological Disaster Prevention and Reduction, Hunan Meteorological Bureau, Changsha, Hunan, China; 3 Xiangtan University& China Unicom (Hunan) Industrial Internet Co., Ltd, China Unicom (Hunan), Changsha, Hunan, China; 4 School of Geography and Information Engineering, China University of Geosciences (Wuhan), Wuhan, Hubei, China; VIT University, INDIA

## Abstract

Cryo-electron micrograph images have various characteristics such as varying sizes, shapes, and distribution densities of individual particles, severe background noise, high levels of impurities, irregular shapes, blurred edges, and similar color to the background. How to demonstrate good adaptability in the field of image vision by picking up single particles from multiple types of cryo-electron micrographs is currently a challenge in the field of cryo-electron micrographs. This paper combines the characteristics of the MixUp hybrid enhancement algorithm, enhances the image feature information in the pre-processing stage, builds a feature perception network based on the channel self-attention mechanism in the forward network of the Swin Transformer model network, achieving adaptive adjustment of self-attention mechanism between different single particles, increasing the network’s tolerance to noise, Incorporating PReLU activation function to enhance information exchange between pixel blocks of different single particles, and combining the Cross-Entropy function with the softmax function to construct a classification network based on Swin Transformer suitable for cryo-electron micrograph single particle detection model (Swin-cryoEM), achieving mixed detection of multiple types of single particles. Swin-cryoEM algorithm can better solve the problem of good adaptability in picking single particles of many types of cryo-electron micrographs, improve the accuracy and generalization ability of the single particle picking method, and provide high-quality data support for the three-dimensional reconstruction of a single particle. In this paper, ablation experiments and comparison experiments were designed to evaluate and compare Swin-cryoEM algorithms in detail and comprehensively on multiple datasets. The Average Precision is an important evaluation index of the evaluation model, and the optimal Average Precision reached 95.5% in the training stage Swin-cryoEM, and the single particle picking performance was also superior in the prediction stage. This model inherits the advantages of the Swin Transformer detection model and is superior to mainstream models such as Faster R-CNN and YOLOv5 in terms of the single particle detection capability of cryo-electron micrographs.

## 1. Introduction

The Fast ParticlePicker [1] proposed in 2017 uses the Faster R-CNN [2] architecture with a simplified region proposal approach to automate human-level particle extraction tasks. However, it uses sliding windows to obtain regions of interest, and its performance mainly depends on the classification network. This search method greatly affects the extraction efficiency and is susceptible to noise. After FastParticlePicker, crYOLO [3] improved the target detection model YOLOv5 [4] for particle extraction in cryo-electron micrographs, aiming to automatically identify particles with high recall and precision, and fully automate the data collection process. However, its experimental section does not mention how the crYOLO framework extracts single particles of other sizes and aspect ratios. Subsequently, many excellent target detection networks emerged, which performed well in natural images (such as Faster R-CNN [5], and Mask Faster R-CNN [6]). In 2019, PIXER proposed a method for locating single particles of a cryo-electron microscope with a low signal-to-noise ratio in the probability density map output from a segmented network, which can be as good in accuracy as the semi-automatic method RELION. In 2020, PARSED [7] proposed an automated and fast deep-learning framework for selecting single particles for cryo-electron micrographs using a fully convolutional network (FCN) as the base network. However, this method requires the generation of known 3D structures of protein molecules and corresponding training sets. In 2019, a keypoint-based detection algorithm, CenterNet [8], was proposed. CenterNet is a fully convolutional network similar to image segmentation networks, with downsampling and upsampling stages, but the final output feature map size is 1/4 of the original image. The network will generate a heatmap, and the value in the heatmap represents the probability that the location belongs to the center point area. The higher the value, the higher the probability of belonging to the center area. CenterNet uses three backbone network structures: Resnet-18, improved DLA-34, and Hourglass-104. The Vision Transformer architecture Swin Transformer, proposed in 2021, builds hierarchical feature maps of images based on linear computational complexity. Swin Transformer [9] constructs hierarchical feature representation through small image slices and hierarchical neighborhood merging. This architecture enables the model to achieve dense prediction tasks similar to U-Net [10] and FPN architectures. Swin Transformer directly applies a Transformer structure to image classification on non-overlapping medium-sized image blocks, while combining with the Swin Transformer model for small target detection. Compared with convolutional networks, it achieves high accuracy in image classification. In 2022, our research team proposed a residual dense neural network Urdnet [11] model based on U-Net, which obtains high-resolution information during upsampling to achieve precise segmentation and positioning. It fills in the underlying information through skip connections to improve segmentation accuracy and has good advantages in cryo-EM image biological image processing.

Therefore, we hope to design an algorithm that conforms to the single particle detection characteristics of cryo-electron micrographs [12] to improve the ability of multi-class single particle location. In order to better adapt to the multi-class cryo-electron micrographs single-particle hybrid detection method, this paper makes improvements based on Swin Transformer, mainly from the aspects of classification function and feature extraction intervention in data pre-processing stage and training stage. A novel multi-class cryo-electron micrographs single-particle hybrid detection model (Swin-cryoEM) based on Swin-transformer was constructed. The main innovations are as follows:

1. Aiming at the problems of low signal-to-noise ratio, high noise intensity and low contrast of cryo-electron micrographs. This model uses Swin Transformer to segment the image into a small piece to input the features of the model training, and adds new data preprocessing processes such as histogram equalization and Gaussian filtering to enhance the image feature information, make up for the deficiency of the training image data set, and strengthen the information interaction of each pixel block to improve image contrast and reduce image noise. The processed image is more suitable for the single particle detection with cryo-electron micrographs to improve the detection accuracy.
2. Aiming at problems such as aliasing effect and poor noise tolerance among multiple classes of cryo-electron micrographs, a local channel attention mechanism model is incorporated into the model in the training stage to perform adaptive regulation on features in channel dimension and spatial dimension to alleviate aliasing effect and reduce the introduction of noise. It also allows the network to quickly focus on important information to increase the network’s tolerance to noise.
3. In order to better solve the problem of poor network generalization and overfitting caused by small datasets due to the fact that multiple classes of images can be classified directly and particles have related similarities, the Cross Entropy function and softmax loss function of multiple classifications are integrated. The gradient descent algorithm can be used to optimize the network parameters to minimize the loss function and avoid the decrease of the learning rate of the mean square error loss function.

## 2. Related work

Cryo-electron micrograph images have the characteristics of low signal-to-noise ratio, low contrast, severe background noise, high impurities, low data volume, difficulty in data annotation, simple image semantics, and fixed structure [13]. Based on the Swin Transformer [14] target model, combined with the single particle detection characteristics of cryo-electron micrographs, this paper constructs a single particle hybrid detection model (Swin-cryoEM) with Swin Transformer as the backbone network. Compared with the Swin Transformer detection model, Swin-cryoEM mainly works in data preprocessing, Swin Transformer algorithm improvement, model overall parameter adjustment, etc.

The main challenge of cryoelectron microscopy is the low signal-to-noise ratio or poor image contrast of cryoelectron microscopy images. Due to the sensitivity of biological samples to radiation, it is difficult to avoid collecting cryo-electron microscopy images with a low signal-to-noise ratio. Therefore, to improve the efficiency of single particle extraction, it is necessary to perform corresponding image preprocessing to enhance the signal-to-noise ratio of the image. The noise in the freeze electron microscope image roughly conforms to the Gaussian distribution, so this paper selects a Gaussian filter to suppress the noise. The intensity of gray pixels directly affects the efficiency of single particle extraction, so this paper uses histogram equalization to improve the image contrast to improve the extraction effect. In addition, to solve the problem of label borders being generally too large, this article scales and adjusts the borders by calculating the center point.

The Swin-cryoEM model combines the characteristics of the MixUp hybrid enhancement algorithm, Enhanced image feature information between various single particles in the preprocessing stage, builds a feature extraction network based on channel self-attention mechanism in the forward network of the Swin Transformer model network, achieving adaptive adjustment of self-attention mechanism between different single particles, increases the network’s tolerance to noise, filter noise information and redundant information from other channels, and integrates the PReLU activation function to realize the nonlinear transformation of the convolution feature map, Strengthen the information exchange of pixel blocks between various types of single particles, and achieve feature extraction of different types of single particles. To avoid the decline of the learning rate of the mean square error loss function, Using a classification network combining the Cross-Entropy function and softmax function to construct a multi-class single particle mixed detection method.

This article will complete the entire process of data preprocessing, model training, and prediction in the dataset of Plasmodium falciparum 80S ribosome (EMPIAR-10028 [15]), micronucleus virus (EMPIAR-10033) [16], human ribosome (EMPIAR-10153 [17]), T20S proteasome (EMPIAR-10057 [18]), Nora virus (EMPIAR-10088 [19]), and TcdA1 (EMPIAR-10089 [20]), and implement a multi-class cryo-electron micrographs single particle mixed detection experiment based on the Swin-cryoEM model.

## 3. Single particle mixed pickup method

### 3.1 Model construction

**Fig 1:**
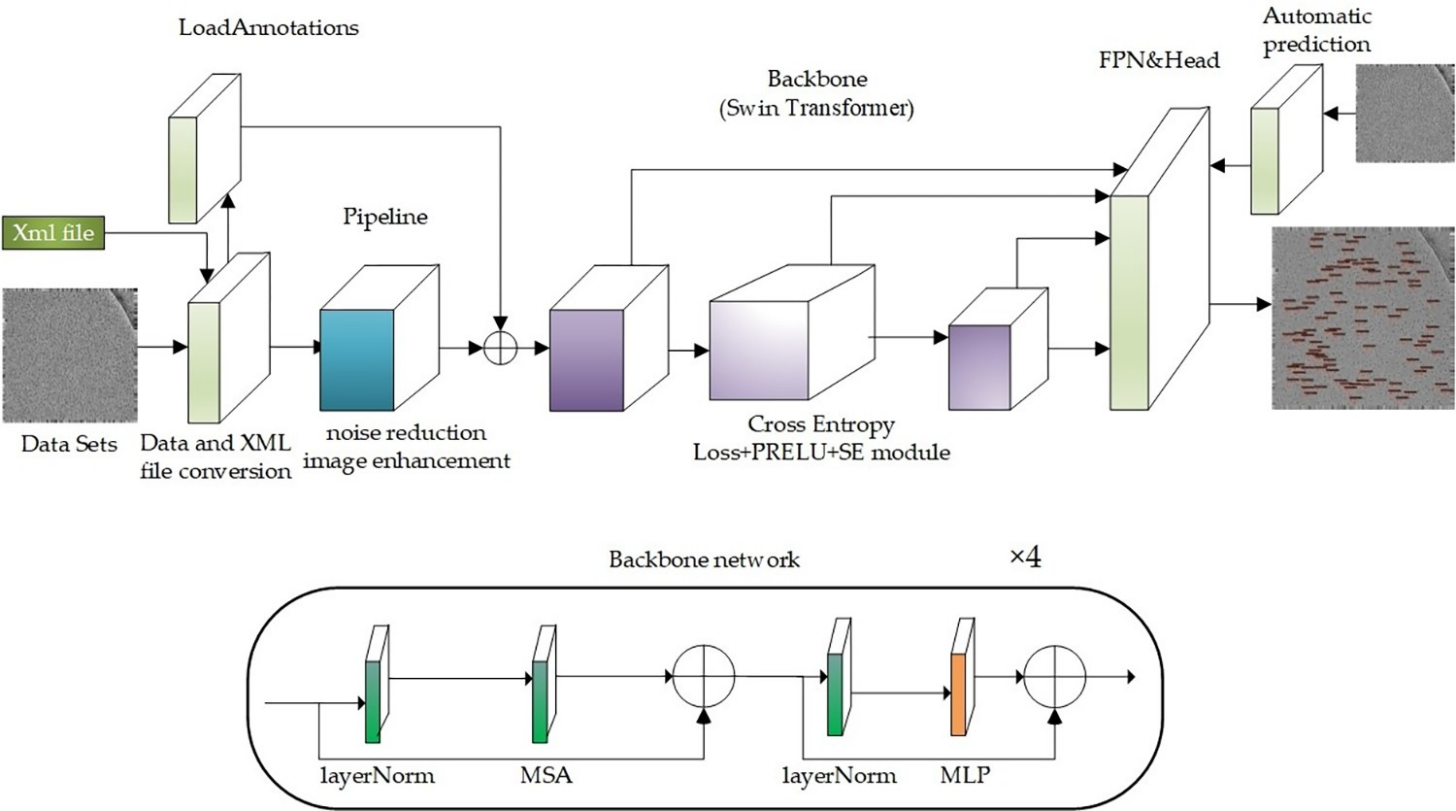
Swin-cryoEM model structure.

This model is based on the Swin Transformer framework and the backbone network is Swin Transformer. Referring to the Swin-B [21] parameter settings, Improve the classification function and feature extraction intervention in the data pre-processing stage and training stage, and retain the original features of Transformer.a suitable cryo-electron micrographs single particle hybrid detection model (Swin-cryoEM) is constructed. The specific Swin-cryoEM structure is shown in Fig 1.

As shown in Fig 1, the overall structure of the Swin-cryoEM model is built based on the Swin Transformer framework. The backbone subnetwork is composed of multi-head autonomous attention mechanism (MSA), multi-layer perceptron (MLP) and two-layer normalized layer (layNorm). The backbone network is iterated through the four-layer backbone subnetwork. In the data pre-processing stage, histogram equalization denoising is added, followed by MixUp image enhancement module. In order to better train single particle features, local channel attention mechanism is added, and the model can complete multi-class classification. Cross Entropy function and softmax loss function of multi-classification are added.The main improvements of the Swin-cryoEM model relative to the Swin Transformer framework are as follows:

1. In the data preprocessing stage, a Gaussian filter is added, and it is slid over the image using a Gaussian kernel. The weighted average of the Gaussian kernel and the corresponding image pixels is calculated as the filtered pixel value. The value of the pixel points in the Gaussian circle region gradually decreases with the distance from the center of the circle, which largely preserves edge information and alleviates distortion after image smoothing. Then, the image is subjected to histogram equalization, which can intuitively reflect the grayscale distribution of the image. If the grayscale distribution is narrow and the spatial domain is large, it indicates that there are few grayscale levels used to express the image, and the image details are not obvious, too dark, or too bright. In terms of data processing, the MixUp hybrid enhancement algorithm is incorporated to enhance the image’s hybrid classification and improve the generalization ability of multi-class target detection data.
2. Add a local channel attention mechanism to the Swin transformer backbone network to adaptively regulate the features between different single particles in the channel dimension, alleviate aliasing effects, reduce the introduction of noise, and allow the network to quickly focus on important information to increase the network’s tolerance to noise.
3. Integrating the Cross-Entropy function with a multi-classification softmax loss function, gradient descent algorithms can be used to optimize network parameters to minimize the loss function and avoid a decrease in the learning rate of the mean square error loss function, To achieve classification and extraction between different single particles.
4. The biggest issue with real datasets is the accuracy of labels. Most cryo-electron microscopy single-particle extraction label files use a four-bit array to store particle position information, representing the upper left corner coordinates and border size, respectively. This article maps labels to corresponding images and finds that the positioning of labels is accurate, but the borders are generally too large. The input label XML file was scaled according to the center point principle for the border. At the same time, based on the coordinates of the upper left corner and the size of the border, the XML file is converted into a JSON file using a conversion algorithm, which conforms to the Coco object detection label data format.
5. Based on the generated training model, an automatic prediction algorithm is designed. Batch prediction mixes cryo-electron micrographs with single particles, reducing a single budget, and realizing automatic operation of the algorithm from three stages of testing, training, and prediction.

### 3.2 Image preprocessing

#### 3.2.1 Noise suppression

Considering real noise as the sum of multiple independent random variables, according to the central limit theorem, the distribution of the mean value of these noises approaches a Gaussian distribution as the total number of noises increases. Therefore, this article selects Gaussian white noise to add noise to clean images to simulate noisy samples. In the training phase, simulated datasets are fused with real datasets for model training. One of the requirements for obtaining high-resolution freeze electron microscope images is to limit the amount of electrons delivered to the sample to minimize the impact of radiation damage. However, a decrease in dose will lead to a corresponding reduction in the signal-to-noise ratio in the image. Low signal-to-noise ratio (SNR) or poor image contrast is a major challenge for cryo-electron micrographs. Due to the sensitivity of biological samples to radiation, it is difficult to avoid collecting cryo-electron micrograph images with low SNR [22]. Therefore, to improve the efficiency of single particle extraction, it is necessary to do corresponding image preprocessing to enhance the image signal-to-noise ratio. The noise in the freeze electron microscope image roughly conforms to the Gaussian distribution, so this paper selects Gaussian filtering to suppress the noise. The Gaussian filter uses a Gaussian kernel to slide on the image and uses the Gaussian kernel as a weight to calculate a weighted average value with the corresponding image pixels as the filtered pixel value. The value of the pixel points in the Gaussian circle region gradually decreases with the distance from the center of the circle, which largely preserves edge information and alleviates the distortion of the image after smoothing. Eq (1) is a two-dimensional Gaussian distribution function [23], where σ Is the standard deviation of the Gaussian distribution. The standard deviation determines the width of the Gaussian filter and also determines the degree to which the image is smoothed. There is an extremely simple relationship between the standard deviation and the degree to which the image is smoothed. The larger the value of the standard deviation, the wider the frequency band of the Gaussian filter, and the smoother the output image. By controlling the standard deviation, a compromise can be achieved between excessively smooth images and noisy images.


$$G(x,y)=\frac{1}{2\pi \sigma^{2}}e^{-\frac{x^{2}+y^{2}}{2\sigma^{2}}}$$ (1)

The process of noise suppression is as follows:

1. Move the filter window to slide it to the image processing area;
2. Multiplying the filter by the pixel values of the area to be processed in the image;
3. Add the multiplied results and take the average value as the new value of the output center pixel.

**Fig 2:**
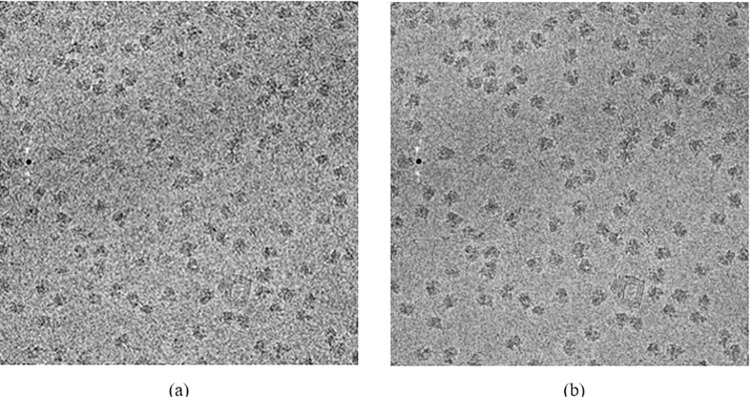
Comparison diagram before and after noise suppression: (a)original; (b)noise suppression.

Fig 2 is a comparison of results before and after Gaussian filtering. It can be seen that after filtering, the particle edges are more obvious, and the image is clearer and smoother.

#### 3.2.2 Histogram equalization

The intensity of gray pixels directly affects the efficiency of single particle extraction, so this article uses histogram equalization to improve image contrast to improve the extraction effect. The histogram of an image can intuitively reflect the grayscale distribution of the image. If the grayscale distribution is narrow and the spatial domain is large, it indicates that there are few grayscale levels used to express the image, and the image details are not obvious, too dark, or too bright. Histogram equalization obtains new pixel values from the pixel distribution in an image through a calculation formula that approximates a uniform distribution, allowing the image to have a larger gray dynamic range and higher contrast while enriching the details of the image. That is, an image with a known gray probability density distribution is transformed into a new image with a uniform gray probability density distribution.

The transformation formula shown in Eq 2 represents the histogram equalization processing principle [24]:

$$S_{k}=T(r_{k})=\sum_{j=0}^{k}p_{r}(r_{i})=\sum_{j=0}^{k}\frac{n_{j}}{n}$$ (2)

**Fig 3:**
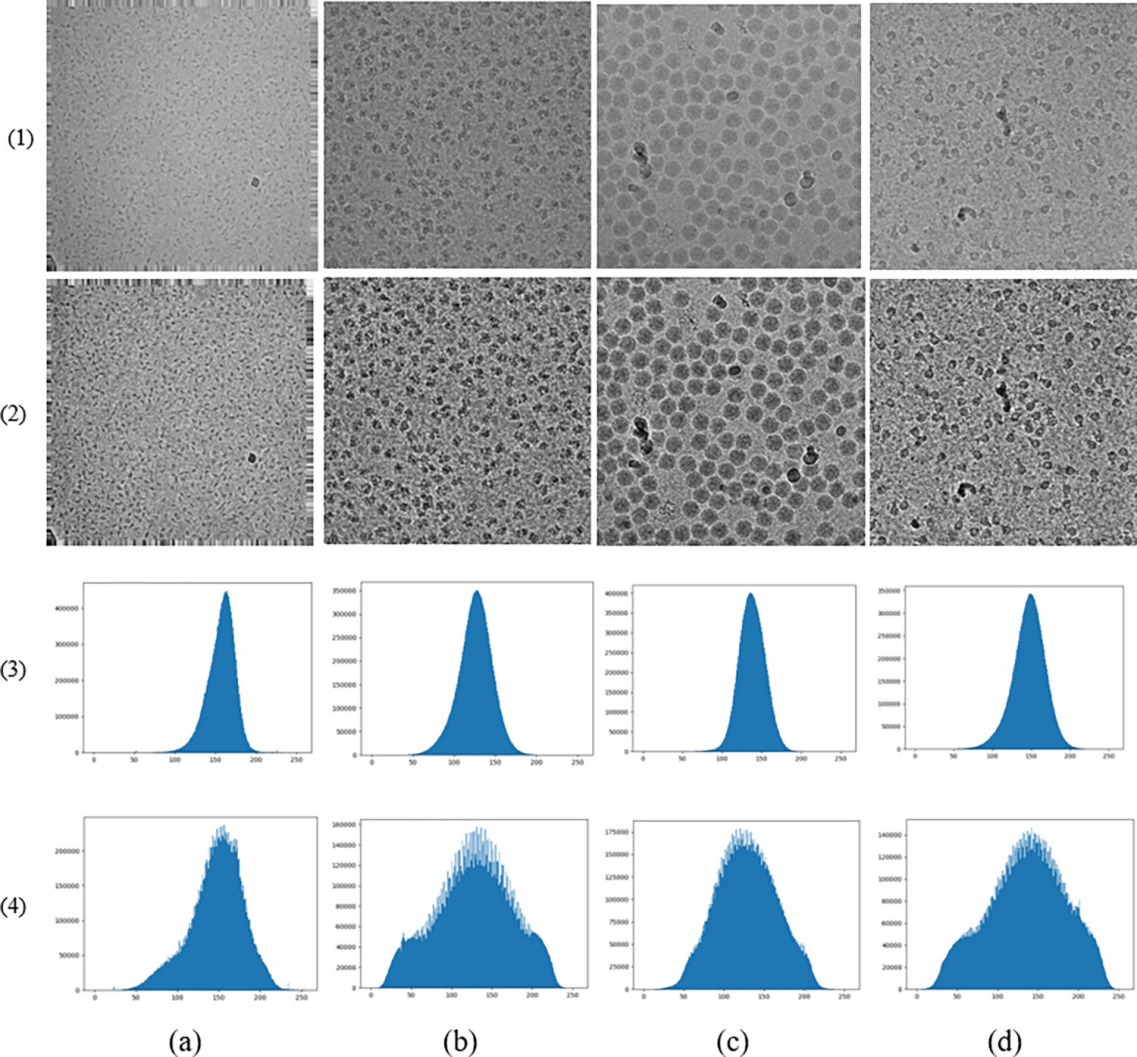
Comparison histogram equalization between multiple categories: (a)EMPIAR-10153; (b) EMPIAR-10017;(c) EMPIAR-10088; (d)EMPIAR-10089; (1)Original;(2)Histogram equalization;(3)expression (1) histogram curve;(4)expression(2) histogram curve.

Where *S*_*k*_ represents the new pixel value to be calculated,n represents the total number of pixels, and *n*_*j*_ represents the number of pixel values j. Fig 3 shows the comparison results before and after histogram equalization. By comparing four different datasets (a), (b), (c), and (d), it is clear that the image after histogram equalization is clearer, with a more pronounced contour, and more pronounced particle edge texture than the original image. At the same time, the histogram of the original image has a wider gray level than the histogram equalization, displaying richer details, and is more conducive to single particle extraction from the freezing electron microscope. The comparison of four different datasets shows that the Swin-cryoEM model has a strong generalization ability.

#### 3.2.3 Incorporating MixUp mixed class enhancement algorithm

Data augmentation is the core of all successful applications of deep learning, from image classification to speech recognition. To improve the generalization ability of multiple types of cryo-electron micrograph image data, this article will incorporate data augmentation algorithms to process cryo-electron micrograph images. Data enhancement mainly uses operations such as rotation, translation, cropping, resizing, flipping, and random erasure to enhance the training dataset, thereby compensating for the shortcomings of the training image dataset, achieving the purpose of expanding the training data and improving the generalization ability of the dataset.

MixUp is a hybrid enhancement algorithm used in computer vision to enhance images, establishing a linear relationship between data enhancement and supervisory signals, generating a powerful regularizer that can improve generalization capabilities. It can mix images from different classes to expand the training dataset. In terms of data enhancement, MixUp has significant advantages over ERM in datasets such as ImageNet-2012 [25] and CIFAR-100.

The Swin-cryoEM model introduces the MixUp data enhancement method, which uses linear interpolation to obtain new extended data, and constructs new training samples and labels using linear interpolation. The MixUp principle formula is shown in (3) and (4) [26]:

$$\overset{ˇ}{x}=ax_{i}+(1-a)x_{j}$$ (3)

$$\overset{ˇ}{y}=ay_{i}+(1-a)y_{j}$$ (4)

Among them, the two data pairs in the Formulas (3) (4) are the training sample pairs (training samples and their corresponding labels) in the original data set. It is the mixing coefficient calculated from the beta distribution with the parameters α and β.

To find the best suitable Swin-cryoEM model for training cryo-electron micrographs images (α, β), select Beta Distribution as (5,1),(2,5),(1,3),(0.5,0.5),(2, 2) and other commonly used (α, β) parameters are trained in the Swin-cryoEM model for cryo-electron micrographs images. The specific experimental results are shown in Table 1.

**Table 1:** The average accuracy of the Swin-cryoEM when MixUp varies with the value of (α, β).

(α, β)	${AP}_{50}^{box}(\%)$	${AP}_{75}^{box}(\%)$
(5,1)	77.1	46.6
(2,5)	84.6	56.9
(1,3)	73	45
(0.5,0.5)	95.5	85.8
(2,2)	90.3	55.7

As shown in Table 1, after multiple iterative training experiments on this model, it is found that the value α = 0.5, β = 0.5, and the average accuracy reaches 95.5%. Therefore, the Swin-cryoEM model (α, β) When the value is (0.5,0.5), the training effect is the best. The target detection model exhibits linearity when processing samples and regions between samples in cryo-electron micrographs single particle dataset, with weak interaction between samples, and insufficient generalization ability in the entire cryo-electron micrographs single particle image. The Swin-CryoEM model adds a MixUp hybrid enhancement algorithm after data input and performs a series of processing such as rotation, translation, cropping, resizing, flipping, and random erasure on cryo-electron micrographs single-particle images. It can represent perceptual objects in different forms, such as angles, pixels, and other transformations. At the same time, it linearly transforms decision boundaries from one class to another, providing a smoother estimate of uncertainty. In the original MixUp text, the average performance of two neural network models was trained using both MixUp and ERM methods on the CIFAR-100 dataset. The two models have the same structure and use the same training process to evaluate the same randomly sampled sample from training data. Models trained with MixUp are more stable in predicting data between training data.

**Fig 4:**
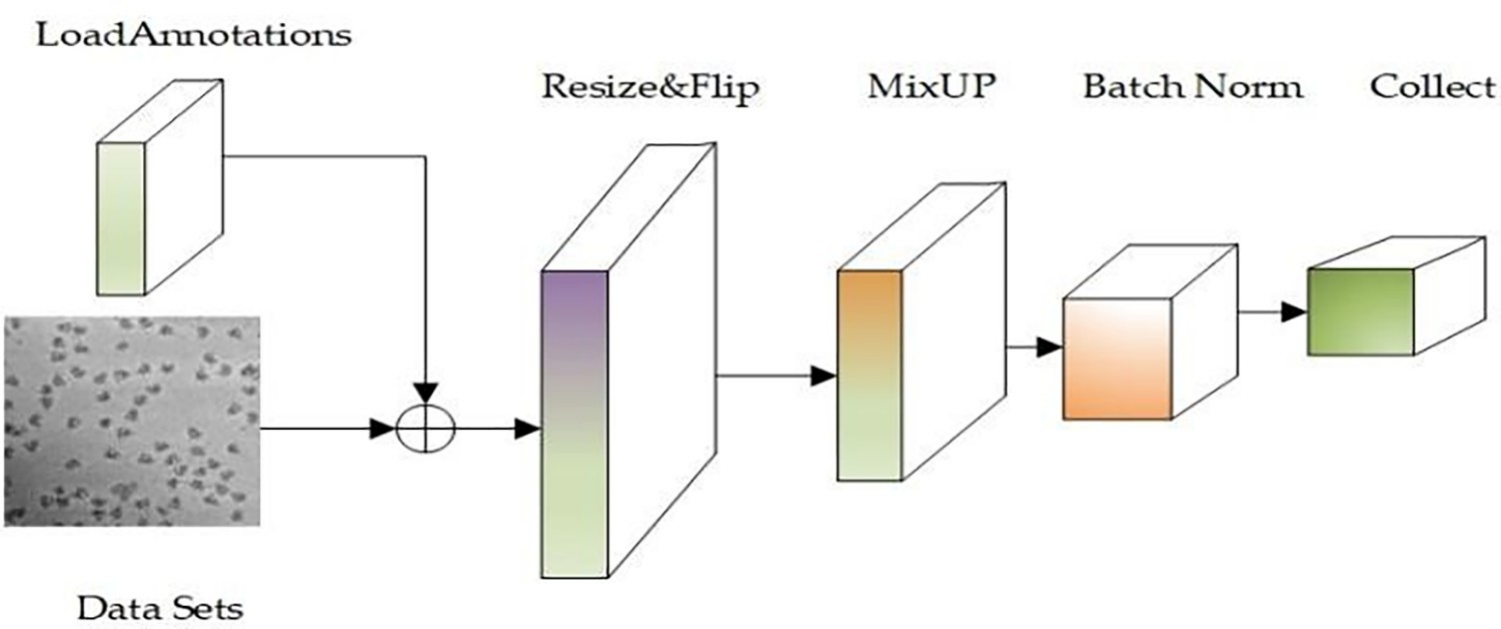
Pipeline process of Swin-cryoEM model.

After incorporating the MixUp mixed class enhancement algorithm into the Pipeline, the Swin-CryoEM model has been experimentally demonstrated to have a stronger generalization ability and higher average accuracy for multi-target detection in the training model. At the same time, it continues the discrete sample space, improves the smoothness in the neighborhood, and compensates for the lack of training image datasets. The pipeline flow sequential operation process is shown in Fig 4.

As shown in Fig 4, this process loads datasets and tag files, randomly flips images, and combines the MixUp algorithm to mix and enhance data between different classes, thereby expanding the training dataset, improving the generalization ability of the data, and enhancing image feature information. Finally, the image will be resized and the processed data will be input to the backbone network for learning through a series of process operations such as normalization and acquisition.

### 3.3 Channel attention based feature extraction mechanism

**Fig 5:**
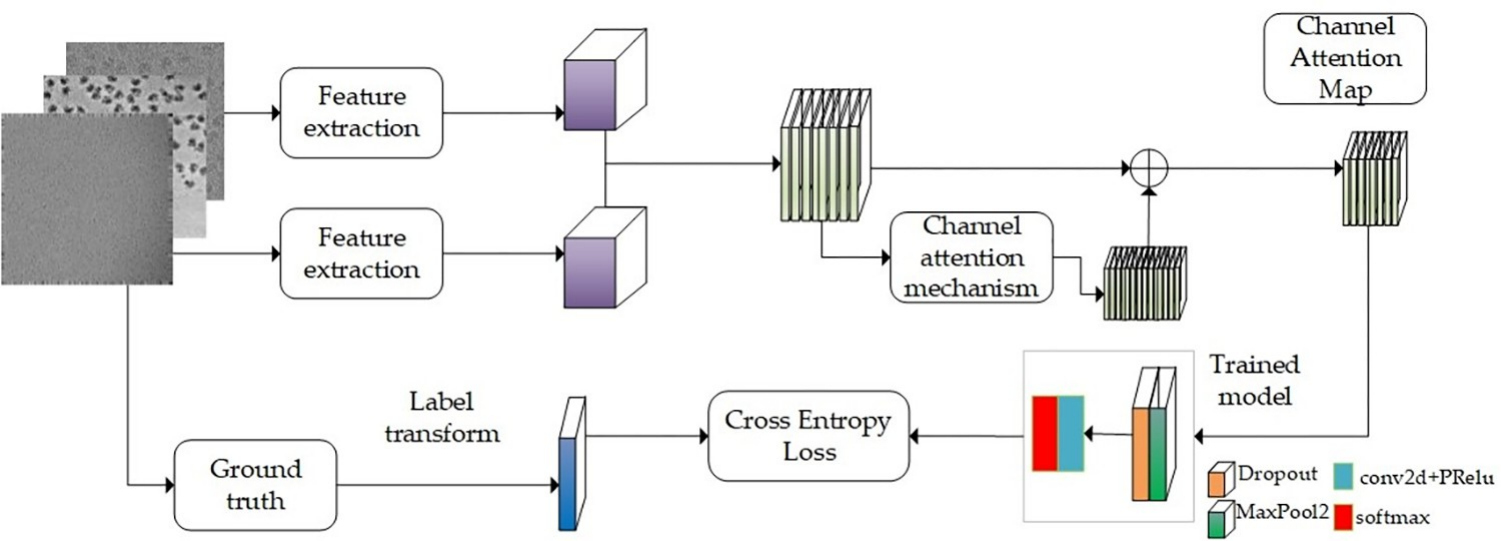
Flow chart of feature extraction and detection methods based on channel attention mechanism.

Squeeze-and-excitation [27] Networks are used for our convolutional layer, which is different from VGG16 [28]. The SE module, also known as the channel attention mechanism, can assign different weights to each channel in the feature map and model the correlation between the channels. Therefore, the model can identify the most relevant channels and filter out noise and redundant information from other channels. The SE module includes two main modules: Squeeze and Excitation. As for squeezing, the global average pool compresses the feature map so that the characteristics of each channel are averaged. After compressing the value, two convolutional layers (with a trainable 1*1 filter bank) generate attention weights (i.e., excitation) for each channel. Finally, the input feature map of each channel is multiplied by the attention weight to obtain the final output feature map, as shown in Fig 5.

To improve the generalization ability of the Swin-cryoEM model and improve the fitting ability of the Swin-cryoEM model, a very small number of parameters are added to the ReLU activation function, and a Parametric Corrected Linear Unit (PReLU) activation function is incorporated. The PReLU [29] activation function adds a linear function to preserve negative input values. Add a PReLU activation function to the forward network, transform nonlinear activation on different channels, enhance the information interaction of each pixel block, maintain a stable convergence rate of the model, alleviate the rapid disappearance of gradients, prevent overfitting risks, reduce network depth, improve feature learning, enhance the scalability of its image, and improve generalization ability.

In addition, extended convolution is also used in the convolution layer, while extended convolution is not used in VGG16. Expanding convolution can expand the receptive field, and the convolution output will contain broader information. Extended convolution is designed for the first and last layers of convolution, with an extension of 2. Other convolution layers are combined with the SE module. The convolution layer includes convolution, batch normalization, and correction linear unit (PReLU) functions. To improve the recognition of single particles by the Swin-cryoEM model and improve the generalization ability of the model, the Swin-cryoEM model incorporates the Cross-Entropy function to achieve mixed extraction of multiple types of single particles. The process of feature extraction and detection based on the channel attention mechanism is shown in Fig 5.

## 4. Experiment and analysis

### 4.1 Data set and processing

This article uses five different particles to construct a training dataset, namely, Plasmodium falciparum 80S ribosome (EMPIAR-10028), micronucleus virus (EMPIAR-10033), human ribosome (EMPIAR-10153), T20S proteasome (EMPIAR-10057), Nora virus (EMPIAR-10088), and TcdA1 (EMPIAR-10089), which contain particles of different sizes and shapes, greatly ensuring the diversity of the training dataset, Enriched the types of single particle features and improved the generalization ability of the algorithm. Most cryoelectron microscopy single particle extraction label files use a four-bit array to store particle position information, representing the upper left corner coordinates and border size, respectively. This article maps labels to corresponding images and finds that the positioning of labels is accurate, but the borders are generally too large. Meanwhile, Freezing electron microscope images have a large size, but are scale and rotation invariant based on the network. To facilitate training, during the dataset production process, all images are scaled to 4096 * 4096 in size. Then each sheet is labeled as an XML file using the labelImg tool. This experiment completes single particle data labeling and converts the XML file into a JSON file through a conversion algorithm, which conforms to the coco target detection label data format.

### 4.2 Model training

This training uses 8 GeForce RTX 3090 graphics cards, CUDA 11.1, CuDNN 8.0.5, and other basic computing frameworks to complete this experiment. Each experiment trained 24 batches and completed the Swin Transformer-based target detection model training, Swin_cryoEM target detection model training, Faster R-CNN target detection model training, YOLOv5 target detection model training, and CenterNet and Urdnet single particle extraction experiments. The experimental data will be analyzed from different perspectives. The training model evaluation indicators mainly follow the Microsoft COCO evaluation system, and the main evaluation indicators are AP (average precision) and AR (average recall) [30].

#### (1) Model training results

To prove that the Swin-cryoEM target detection method is more suitable for single particle extraction tasks under freezing electron microscopy. This article uses the same dataset to train the Faster R-CNN, CenterNet, Urdnet, YOLOv5, Swin Transformer(Swin Transformer stands for original model), and Swin-cryoEM models.

Table 2 shows the comparison of relevant indicators for single particle extraction using Cryo-electron micrographs in Faster R-CNN, CenterNet, CenterPicker, YOLOv5, Swin Transformer, and Swin-cryoEM target detection models. It can be seen that when the IOU is 0.5, the Swin-cryoEM model AP reaches 95.5%, while the Swin Transformer model AP is 81.5%. The Swin-cryoEM model AP is about 14% higher than the Swin Transformer model, and about 49.4% higher than the Faster R-CNN model, Compared to YOLOv5, it is also about 18.8% higher. The AP of the CenterNet model and Urdnet model based on the center point cryo-electron micrographs single particle extraction algorithm is 64.7% and 87.4%, respectively, which is about 30.7% and 8% lower than that of the Swin cryoEM model, respectively; When the IOU is 0.75, the Swin-cryoEM model AP reaches 85.8%, while the Swin Transformer model AP is 69.5%. The Swin-cryoEM model AP is about 16.3% higher than the Swin Transformer model, and about 61.8% higher than the Faster R-CNN model. Based on the center point cryo-electron micrographs single particle extraction algorithm, the CenterNet model and Urnet model AP are 57.8% and 75.9%, respectively, which are about 28% and 9.9% lower than the Swin-cryoEM model AP. From the above results, it can be seen that the Swin-cryoEM model AP is significantly superior to Faster R-CNN, CenterNet, CenterPicker, YOLOv5, and Swin Transformer in detecting single particle targets in cryo-electron micrographs.

**Table 2:** Comparison of model training results.

Model	AP	AP^50^	AP^75^	AR
Faster R-CNN	0.248	0.461	0.240	0.338
CenterNet	0.367	0.647	0.578	0.505
Urdnet	0.620	0.874	0.759	0.678
YOLOv5	-	0.767	-	-
Swin Transformer	0.602	0.815	0.695	0.677
**Swin-cryoEM**	**0.65**	**0.955**	**0.858**	**0.714**

In the single-particle extraction training in Table 2, the AR of models Faster R-CNN, CenterNet, Urdnet, Swin Transformer, and Swin-cryoEM were 33.8%, 50.5%, 67.8%, 67.7%, and 71.4%, respectively, indicating the superiority of Swin-cryoEM.

In contrast, Swin-cryoEM detects more particles, and the size and position of the bounding box are very accurate. Swin-cryoEM can adapt to particle extraction tasks with different particle sizes. The experimental results also prove that the Swin-cryoEM target detection network is more suitable for the single particle extraction task of cryo-electron micrograph images.

#### (2) Performance comparison analysis

**Fig 6:**
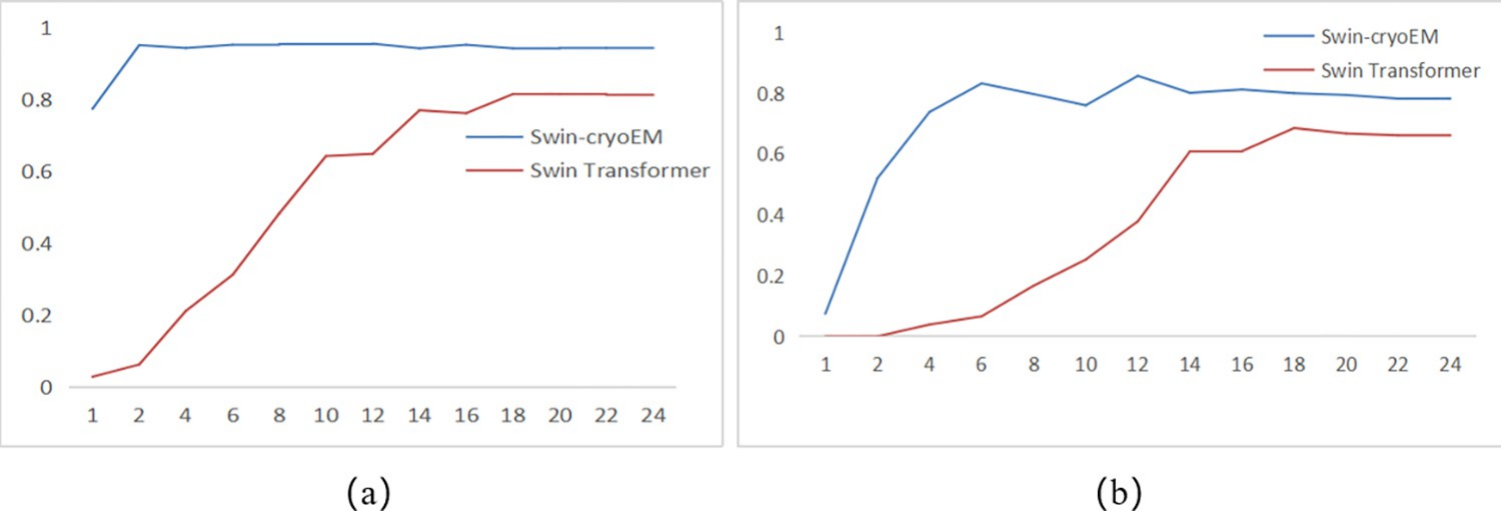
The trend of IOU value AP between the Swin-cryoEM model and Swin Transformer model: (a) IOU = 0.5;(b) IOU = 0.75.

This article is mainly based on the improvement of the Swin Transformer model to complete the construction of the Swin-cryoEM model. In addition to verifying the maximum AP index and AR index of the model, it is also necessary to verify the stability and convergence of the model during the training process. In Fig 6, compare different IOU values for the Swin-cryoEM model and the Swin Transformer model, and observe the trend of the AP.

From the curve trend of Figure (a) in Fig 6, it can be found that the Swin-CryoEM model has rapidly grown to the highest value, approximating a straight line, with small fluctuations, and has consistently been higher than the average accuracy of the Swin Transformer model. This indicates that the Average Precision of the Swin-cryoEM model is superior to the Swin Transformer model. From the third batch to the 24th batch, the Swin-cryoEM model maintains a relatively stable AP value. From the experimental comparison, it can be seen that the Swin-cryoEM model has good stability and convergence.

From the curve trend of Figure (b) in Fig 6, it can be found that the AP value of the Swin-CryoEM model has increased rapidly, approximately linearly, and fluctuated slightly, consistently higher than the average accuracy of the Swin Transformer model. This indicates that the Average Precision of the Swin-cryoEM model is superior to the Swin Transformer model. From the 14th to 24th batches, the Swin-cryoEM model maintains a relatively stable AP value. From the experimental comparison, it can be seen that the Swin-cryoEM model has good stability and convergence.

**Fig 7:**
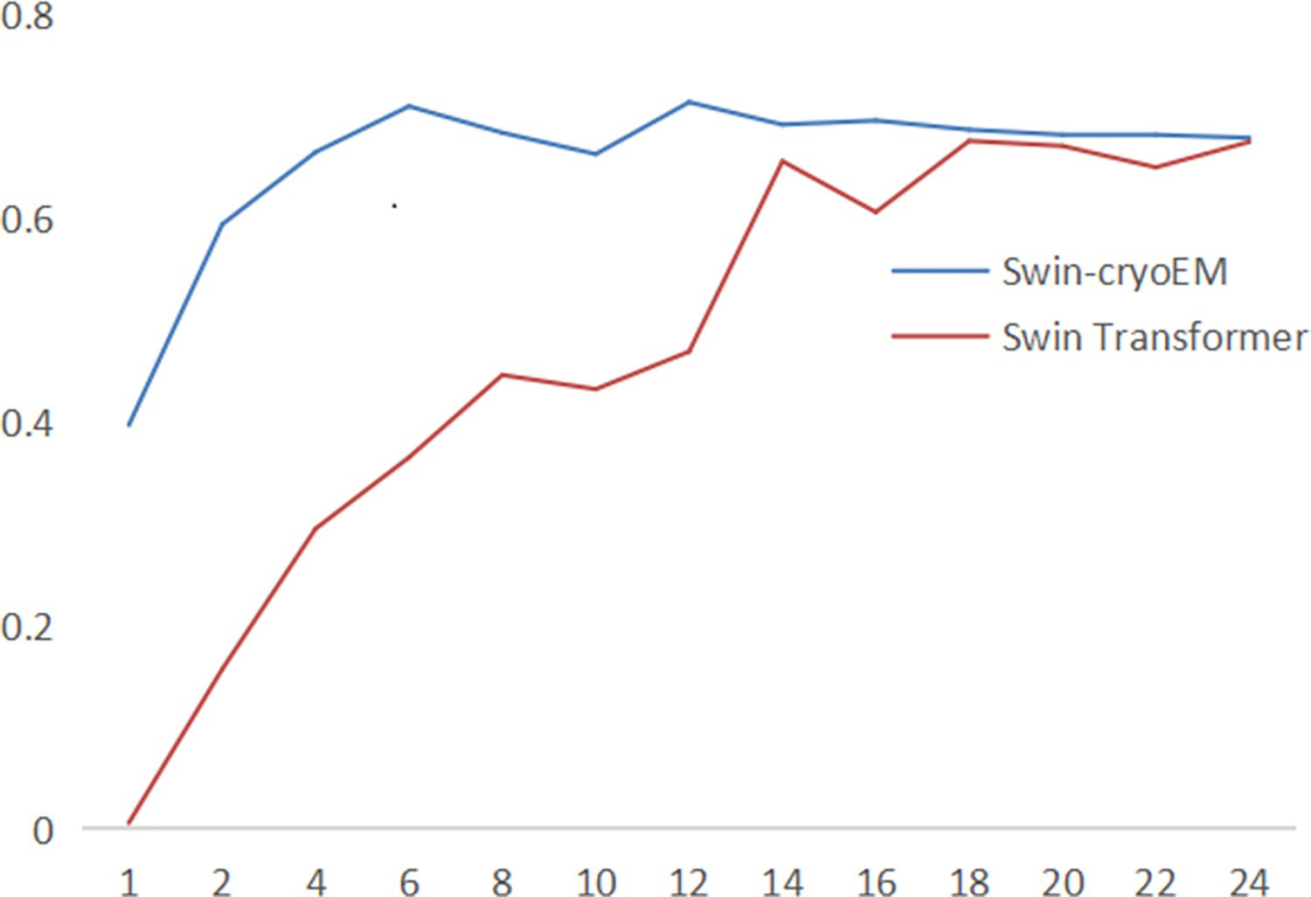
Comparison of AR between the Swin-cryoEM model and Swin Transformer model.

In Fig 7, observe the trend of AR during the training process of the Swin-cryoEM model and the Swin Transformer model.

In Fig 7, it can be found that the average recall rate of Swin-CryoEM in 1 to 6 batches has increased rapidly, with approximate linear growth and small fluctuations. Compared to the Swin Transformer, it has stronger stability. At the same time, the overall average recall rate of Swin-cryoEM during the entire training process is higher than that of Swin Transformer, with a maximum of 0.714. Compared to the Swin Transformer model, Swin-cryoEM has stronger learning performance and also exhibits stronger convergence and stability.

**Fig 8:**
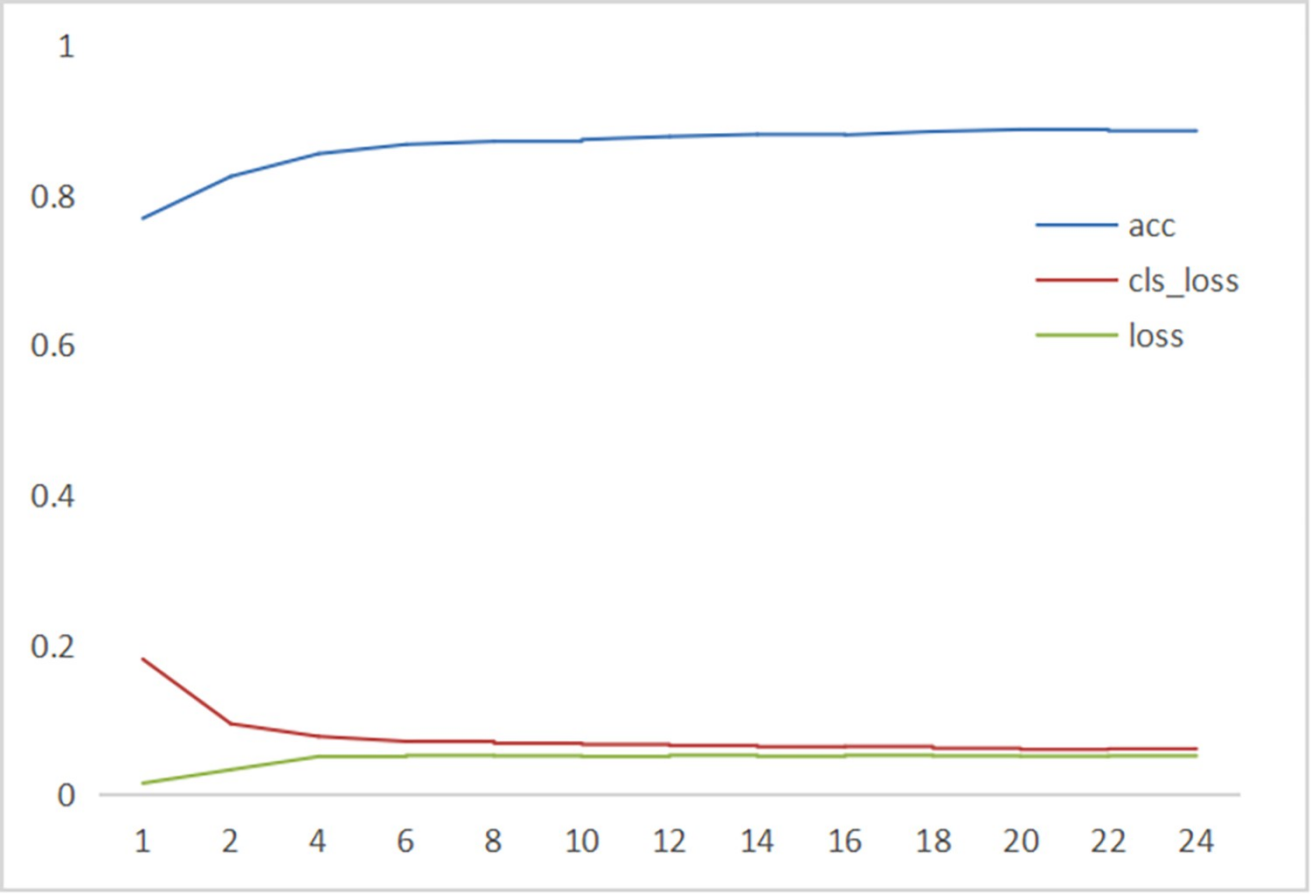
Swin-cryoEM model acc,cls_Loss, and loss analysis.

To further verify the stability and convergence of the model, observe the changes in loss and accuracy during the training process. In Fig 8, extract acc (accuracy), loss (basic loss), and cls_ Loss (cross-entropy loss) observe its trend during training.

In Fig 8, it is evident that the three curves converge faster, with almost no shocks after convergence, and the curve is smoother. As shown in Fig 4, the Swin-cryoEM model can filter the impact of noise on the network by introducing an attention mechanism, enabling the network to quickly find the correct direction during training, making the model training more stable and have better convergence, and achieving better accuracy.

### 4.3 Model prediction results

Table 3 shows that in the dataset b-galactosidase (EMPIAR-10017 [31]), to further verify the ability of the trained convergent model to predict single particles, this paper conducts further prediction experiments by comparing the detected particle coordinates with the labels. If the distance between the extracted particle coordinates and the label coordinates is less than 30% of the particle size in the test, the particle is considered as the correctly extracted particle, To verify the stability and accuracy of each model. The particle coordinates in the EMPIAR-10017 dataset of the prediction experiment were manually selected by Richard Henderson, including a total of 84 microscopic images with a resolution of 4096*4096, and 40862 particle coordinates are included. This article uses an accuracy rate to evaluate the performance of the network. Accuracy can measure the correlation between prediction results and labels, as well as the ability of network detection to distinguish positive samples.

**Table 3:** Comparative experiment results.

Methods	Swin-cryoEM	Urdnet	CenterNet	Swin Transformer	Faster R-CNN	YOLOv5
Picked particles	**44163**	48676	48656	48991	46890	46261
True positive	**39170**	41158	30180	40437	26165	33154
False positive	**4993**	7518	18476	8554	20725	13107
Precision rate(%)	**88.7**	84.6	62	82.5	55.8	71.67

As shown in Table 3, in the extraction of EMPIAR-10017, the Swin-cryoEM model detected 44163 particles, showing better recognition ability and fault tolerance among other models. The correct particles were 39170, which was also better than other models. In addition, the accuracy was 88.7%, confirming the high performance and strong superiority of the Swin-cryoEM model in particle-picking tasks.

**Fig 9:**
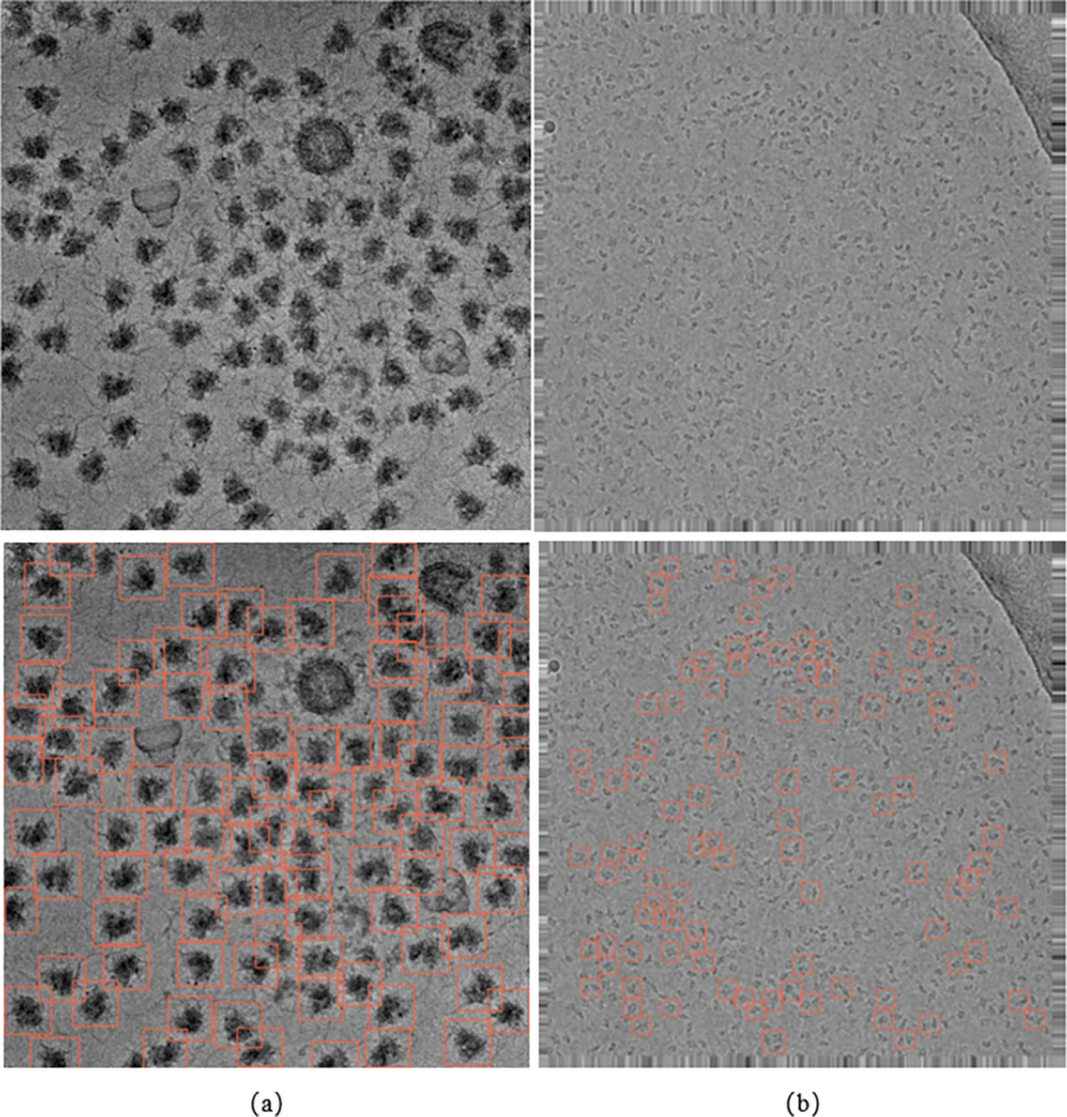
Test results of different types of particles in EMPIAR-10153 and EMPIAR-10017: (a) dataset human ribosomes (EMPIAR-10153); (b) Data set b-galactosidase (EMPIAR-10017).

To verify that the picking up of multiple types of cryo-electron micrographs single particles exhibits good adaptability in the Swin-cryoEM model and can complete the ability to pick up multiple types of cryo-electron micrographs single particles, a prediction experiment was conducted by selecting particles with relatively large differences between EMPIAR-10153 and EMPIAR-10017. The prediction experiment results are shown in Fig 9.

From the data set Human Ribosome (EMPIAR-10153) shown in Fig 9, based on the prediction results, the single particles needed to be picked up in the experiment were detected, while the incorrect particles were also excluded. From the detection effect, the human ribosomal particles in EMPIAR-10153 were almost detected, fulfilling the experimental pickup requirements. (b) In the figure, the detection of b-galactosidase particles was completed through the dataset of b-galactosidase (EMPIAR-10017). The experimental prediction results excluded many non-b-galactosidase particles. In such small particles, the detection of b-galactosidase particles was achieved, achieving the desired effect for the experiment. According to graphs (a) and (b), the Swin-cryoEM model exhibits good adaptability in the prediction process for particles of different sizes, and can accurately pick up particles, proving that the Swin-cryoEM model can complete mixed detection of multiple types of cryo-electron micrographs single particles.

### 4.4 Discussion

Through the comparative analysis of two different experimental stages in sections 4.2 and 4.3 of the experimental results, the Swin-CryoEM model has achieved good results in terms of Average Precision, accuracy, and recall on cryo-electron micrograph images, while outstanding performance in stability and convergence. From the prediction results, it can complete the mixed detection of multiple cryo-electron micrographs and single particles.

From Table 1, this article selects the Beta Distribution commonly used(α, β) Comparative experiments with fixed parameters have found that the parameters in MixUp α = 0.5 and β = 0.5 In the Swin-cryoEM model, the Average Precision is relatively higher, and in applications where other types of images or target detection are of different interest, you can try using Beta Distribution to be very useful (α, β) Relevant experiments were conducted with fixed parameters.

This article is based on the improvement of the Swin Transformer model, which adds noise suppression and histogram equalization in the data preprocessing stage while introducing a local attention mechanism. When applied to other types of images or backgrounds with different interests in target detection, other preprocessing methods can be considered, as well as spatial attention mechanisms and mixed attention mechanisms.

Currently, only small particles of Plasmodium falciparum 80S ribosome (EMPIAR-10028), micronucleus virus (EMPIAR-10033), human ribosome (EMPIAR-10153), T20S proteasome (EMPIAR-10057), Nora virus (EMPIAR-10088), and TcdA1 (EMPIAR-10089) have been trained. Future research can attempt large particle cryo-electron micrograph image mixing training. In the future, it can be considered to cross-train all single particles into the network through a single channel, so that MRC images can be applied to more network models, such as reinforcement learning.

The verification experiment of this model is mainly aimed at the multi-class picking of small particles, which has certain limitations. The performance of the large-particle picking model is the main direction of subsequent research, and the fusion verification with three-dimensional reconstruction needs to be strengthened.

## 5. Conclusion

This model uses Swin Transformer to segment the image into a small piece to input the features of the model training, and adds new data preprocessing processes such as histogram equalization and Gaussian filtering to enhance the image feature information, make up for the deficiency of the training image data set, and strengthen the information interaction of each pixel block to improve image contrast and reduce image noise. Make the processed image more suitable for single particle detection with cryo-electron micrographs. The local channel attention mechanism model was integrated into the training stage model to perform adaptive regulation on features in channel dimension and spatial dimension, alleviate aliasing effect, reduce the introduction of noise, and allow the network to quickly focus on important information to increase the network tolerance to noise. Incorporating the Cross Entropy function, the multi-class softmax loss function, the gradient descent algorithm can be used to optimize the network parameters so as to minimize the loss function and avoid the decrease in the learning rate of the mean square error loss function.

Based on the experiment of single particle image data set with cryo-electron micrographs, from the training results, The maximum Average Precision of SWin-Cryoem model is higher than that of Faster R-CNN, CenterNet, CenterPicker, YOLOv5 and Swin Transformer. The experiment shows that SWin-Cryoem model inherits the advantages of Swin Transformer, and has some advantages in stability and robustness with the increase of training batches. At the same time, the single particle image target detection method with cryo-electron micrographs can train a higher Average Precision. From the prediction results, it can complete the detection of multiple types of single particle mixture with cryo-electron micrographs. Meanwhile, compared with other detection methods compared in this paper, relatively more particles are correctly detected. In the process of ablation experiment, training model experiment, prediction model experiment and other experiments, The Swin-cryoEM model has the advantage of large cryo-electron micrographs single-particle detection pick.

In summary, the Swin-cryoEM model used in this article has more advantages, achieving the expected results of the improved algorithm in this article. The Swin-cryoEM algorithm inherits the structure of the Swin Transformer model and combines the local attention mechanism, MixUp data enhancement algorithm, and Cross Entropy function to achieve multi-class cryo-electron micrographs single particle mixed detection. The Swin-CryoEM algorithm solves the problem of good adaptability in picking up multiple types of cryo-electron micrographs single particles and improves the accuracy and generalization ability of the single particle picking method. Provide high-quality data support for single particle 3D reconstruction. This method can be extended to ground observation data with simple semantics and single features to construct a spatial observation dataset for network training. At the same time, the new visual crack width measurement based on the dual scale feature of the backbone in subsequent work is indeed a new attempt direction, which can select high-quality and standardized single particles based on the width measurement, and can also serve as an important indicator for single particle classification.
